# Zoonotic Transmission of Diphtheria from Domestic Animal Reservoir, Spain

**DOI:** 10.3201/eid2806.211956

**Published:** 2022-06

**Authors:** Andreas Hoefer, Silvia Herrera-León, Lucas Domínguez, Maria Ordobás Gavín, Beatriz Romero, Ximena Belen Araujo Piedra, Cristina Sobrino Calzada, María José Uría González, Laura Herrera-León, Case Study Investigation Group

**Affiliations:** European Public Health Microbiology Training Programme, Stockholm, Sweden (A. Hoefer, S. Herrera-León);; Instituto de Salud Carlos III, Madrid, Spain (A. Hoefer, S. Herrera-León, L. Herrera-León);; Universidad Complutense de Madrid, Madrid (L. Domínguez, B. Romero);; Dirección General de Salud Pública, Madrid (M. Ordobás Gavín);; Hospital Universitario del Sureste, Madrid (X.B. Araujo Piedra, C. Sobrino Calzada);; Laboratorio Br Salud Ut, Madrid (M.J. Uría González)

**Keywords:** diphtheria, zoonoses, bacteria, bacterial infections, *Corynebacterium ulcerans*, Spain

## Abstract

Toxigenic *Corynebacterium ulcerans* is as an emerging zoonotic agent of diphtheria. We describe the zoonotic transmission of diphtheria caused by toxigenic *C. ulcerans* from domestic animals in Spain, confirmed by core-genome multilocus sequence typing. Alongside an increasing number of recent publications, our findings highlight the public health threat posed by diphtheria reemergence.

Diphtheria has been increasing in relevance because of increasing individual travel and surges in mass relocation events of refugees, asylum seekers, and immigrants from countries where diphtheria remains endemic (*1*–*3*). These importation events, in combination with growing vaccine hesitancy in nonendemic countries, give diphtheria a high potential for reemergence.

Toxigenic *Corynebacterium ulcerans*, an agent of diphtheria, has frequently been identified in domesticated animals such as cats, dogs, and pigs in which zoonotic transmission has been demonstrated (*4*–*7*). Toxigenic *C. ulcerans* has also been identified in wild animals such as ferrets, boars, and deer (*8*). This natural reservoir of *C. ulcerans,* in both wild and domesticated animals, constitutes a major public health threat.

## The Case Report

On February 23, 2019, a 60-year-old man visited the emergency department of the Hospital Universitario del Sureste in Madrid, Spain. At examination, he was found to have odynophagia, dysphonia, and a whitish membrane in the oropharynx. He visited this emergency department several times during February 26–March 5. On March 5, an emergency department doctor took a pharyngeal exudate sample and sent it to Spain’s National Centre of Microbiology (CNM), and the patient was started on a course of clarithromycin on March 6.

CNM received the swab on March 7. On March 10, laboratory cultures confirmed that the throat swab contained *C. ulcerans* and was positive for the *tox* gene on PCR (*9*). The hospital then contacted the patient, who was recovering at home, and requested his immediate hospitalization for treatment and isolation. The patient complied and was started on a 12-hour course of intravenous clarithromycin. Administering antitoxin was ruled out because the patient responded well to antibiotics. 

On March 11, the National Directorate of Epidemiologic Services initiated an outbreak investigation. CNM sent a culture to the Centre for Reference on Diphtheria and Streptococcal Infections (part of the United Kingdom’s Health Security Agency) for toxigenicity testing, where the sample was confirmed as toxigenic by ELEK test on March 18 (*10*). Subsequent samples, taken on March 21 and 22, were negative for *C. ulcerans*, and the patient was released. While in the hospital, the patient also was revaccinated for diphtheria.

The Regional Epidemiologic Services of Madrid (RESM) conducted a survey, which confirmed that no exposure to conventional sources of infection or recent overseas travel had occurred. The case-patient and his partner live in relative isolation ≈8 km from a small urban center and are not associated with any agriculture activities. No record of a recent diphtheria booster dose was found for the case-patient or his partner. The case-patient owns 2 cats and 3 dogs, and he is known to regularly feed stray cats that frequent his estate.

RESM conducted contact tracing as indicated by national guidelines (*11*). A risk assessment identified 2 close contacts (considered high-risk) and 20 further contacts (considered moderate-risk). All 22 contacts were tested, and no *C. ulcerans* was identified. The asymptomatic household contact, the case-patient’s partner, received prophylactic azithromycin, and diphtheria vaccine was administered. The second high-risk contact was the attending physician who performed the physical examination without the personal protective equipment required when treating a patient with an active case of diphtheria. The physician’s vaccination history was confirmed, and prophylactic azithromycin was administered. The 20 moderate-risk contacts (including 1 domestic assistant and 19 hospital staff members) all had their vaccination coverage confirmed and were briefed on recognizing potential symptoms.

In adherence with World Health Organization and national protocols, RESM requested Animal Health Services of Madrid to investigate the animals in contact with the human case (*12*). This investigation was performed by the VISAVET Health Surveillance Centre. On March 18–19, nasal, pharyngeal, and conjunctival swabs were collected from the 2 cats (CAT1 and CAT2) and 3 dogs (DOG1, DOG2, and DOG3) that lived with the human case-patient. Three isolates from nasal swab specimens were obtained during selective culturing, 1 from CAT1, 1 from CAT2, and 1 from DOG1 (European Nucleotide Archive accession nos. ERR6177889, ERR6177890, and ERR6177890, respectively); the 2 other dogs tested negative. All isolates were identified as *tox* gene–bearing *C. ulcerans* by PCR and whole-genome sequencing. All 3 animals that had tested positive were placed in isolation by the Central Animal Shelter of the Madrid Community and treated with amoxicillin for 15 days. The animals were retested, and all the swab specimens collected were negative, at which point, the animals were returned to their owner. Workers at the animal shelter were briefed on biosafety measures and management of the infected animals. Contact tracing of the cats was performed, and 4 stray cats were captured and tested; all were negative for *C. ulcerans*. All domestic and stray animals tested were asymptomatic.

All microbiologic procedures were conducted by NCM in accordance with World Health Organization guidelines (*13*). VISAVET identified the isolates by using matrix-assisted laser desorption/ionization time-of-flight mass spectrometry.

CNM purified the genomic DNA from *C. ulcerans* isolates with the DNeasy Blood and Tissue Kit (QIAGEN, https://www.qiagen.com). Libraries were prepared by using Nextera XT DNA Library Preparation Kit and sequenced on a MiSeq platform by using version 3 reagents for 2 × 300 paired-end libraries (both from Illumina, https://www.illumina.com). Multilocus sequence typing (MLST) based on 7 housekeeping *loci* extracted from the next-generation sequencing data identified the human and animals isolates as sequence type (ST) 514 (*14*). Next-generation sequencing–derived core-genome MLST comprising 2,170 target loci revealed no allelic differences between the human (European Nucleotide Archive accession no. ERR4880084) and CAT2 strains, whereas CAT1 harbored 1 allelic difference (*4*). The strain from DOG1 had 4 allelic differences from the human and CAT2 strains, indicating a close relationship (*4*) (Figure). Zoonotic *C. ulcerans* collected from the human, dog, and cat exhibited a high degree of similarity, whereas epidemiologically nonrelated strains differed by thousands of single-nucleotide polymorphisms from each other (data not shown) (*6*,*8*).

**Figure:**
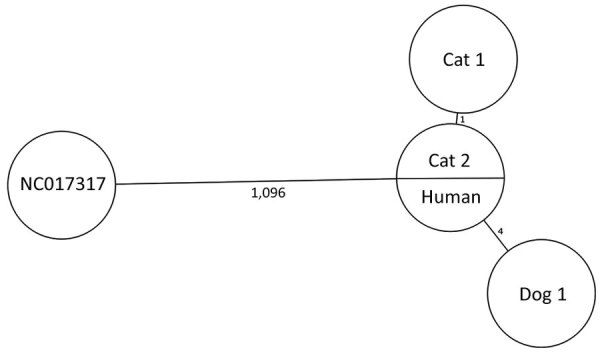
Core-genome multilocus sequence typing–based minimum spanning tree of all toxigenic *Corynebacterium ulcerans* strains associated with the zoonotic transmission of diphtheria in Spain in 2019 compared with reference strain NCTC_NC017317 from GenBank. Branches are labeled with the number of allelic differences between strains. European Nucleotide Archive accession numbers: human strain, ERR4880084; CAT1 strain, ERR6177889; CAT2 strain, ERR6177890; DOG1 strain, ERR6177891.

## Conclusions

The number of cases of diphtheria caused by toxigenic *C. ulcerans* with an epidemiologic link to domestic animals is small but rising (*4*). Under the scope of the One Health initiative, the collaboration between human and animal public health authorities was essential to identify the origin of this case. This case report highlights the sustained risk posed by zoonotic toxigenic *C. ulcerans* reservoirs in peridomestic and domestic animals. Given the high degree of conservation between the human and animal strains, a zoonotic transmission has certainly occurred in this instance. Although the captured stray cats tested negative for *C. ulcerans*, only a small portion of the stray cats in contact with the domesticated animals could be tested. The actual number of cats that were in direct contact with the domesticated animals is unknown. Currently, *C. ulcerans* is not a notifiable organism if it is detected in animals (*11*). To mitigate the future public health burden of toxigenic *C. ulcerans* from animal reservoirs, its declaration should be considered as part of the national surveillance guidelines.

Feline, canine, and porcine zoonotic transmission of toxigenic *C. ulcerans* has been previously supported by findings of the same ST (derived from MLST) in the suspected animal and the epidemiologically linked human case or cases (*7*). The ST identified in this study (ST514) was previously described in an isolate from a 59-year-old man with cutaneous lesions in France in 2005 (*14*).

Vaccinations against diphtheria are offered at 2, 4, and 11 months of age in Spain, with booster doses at 6, 14, and >65 years of age. Earlier administration of boosters may need to be considered because levels of antibodies may not be sufficient to prevent the disease in older persons who are <65 years of age (*15*).
